# Genome-wide sequence-based prediction of peripheral proteins using a novel semi-supervised learning technique

**DOI:** 10.1186/1471-2105-11-S1-S6

**Published:** 2010-01-18

**Authors:** Nitin Bhardwaj, Mark Gerstein, Hui Lu

**Affiliations:** 1 Bioinformatics Program, Department of Bioengineering, University of Illinois at Chicago, Chicago, IL 60607, USA; 2 Program in Computational Biology and Bioinformatics, Yale University, Bass 432, 266 Whitney Avenue, New Haven, CT 06520, USA; 3 Department of Molecular Biophysics and Biochemistry, Yale University, Bass 432, 266 Whitney Avenue, New Haven, CT 06520, USA; 4 Department of Computer Science, Yale University, Bass 432, 266 Whitney Avenue, New Haven, CT 06520, USA

## Abstract

**Background:**

In *supervised learning*, traditional approaches to building a classifier use two sets of examples with pre-defined classes along with a learning algorithm. The main limitation of this approach is that examples from both classes are required which might be infeasible in certain cases, especially those dealing with biological data. Such is the case for membrane-binding peripheral domains that play important roles in many biological processes, including cell signaling and membrane trafficking by reversibly binding to membranes. For these domains, a well-defined *positive *set is available with domains known to bind membrane along with a large *unlabeled *set of domains whose membrane binding affinities have not been measured. The aforementioned limitation can be addressed by a special class of *semi-supervised *machine learning called *positive-unlabeled (PU) *learning that uses a positive set with a large unlabeled set.

**Methods:**

In this study, we implement the first application of *PU-learning *to a protein function prediction problem: identification of peripheral domains. *PU-learning *starts by identifying reliable negative (*RN*) examples iteratively from the unlabeled set until convergence and builds a classifier using the positive and the final *RN *set. A data set of 232 positive cases and ~3750 unlabeled ones were used to construct and validate the protocol.

**Results:**

Holdout evaluation of the protocol on a left-out positive set showed that the accuracy of prediction reached up to 95% during two independent implementations.

**Conclusion:**

These results suggest that our protocol can be used for predicting membrane-binding properties of a wide variety of modular domains. Protocols like the one presented here become particularly useful in the case of availability of information from one class only.

## Background

Formally, a typical classification problem can be stated as follows: given training data {(*x*_1_, *y*_1_), ...,(*x*_*n*_, *y*_*n*_)}, produce a classifier *f*: *X *→ *Y *which maps an object *x *∈ *X *to its classification label *y *∈ *Y *[1]. The **x**_*i *_values are typically vectors of the form <*x*_*i*, 1_, *x*_*i*, 2_, ..., *x*_*i*, *n*_>. Given new **x **values, the classifier predicts the corresponding *y *values. For example, if the problem is that of filtering spam, then *x*_*i *_is some representation of an email (such as the subject, body, etc.) and *y *is either "Spam" or "Non-Spam". This form of machine learning is called as *supervised learning *where the aim is to establish a rule whereby a new observation can be classified into one of the existing known classes. Another class of machine learning is the *unsupervised learning *where a set of observations are given with the aim of establishing the existence of classes or clusters in the data and the prior distribution of the data is usually not known.

**Figure 1:**
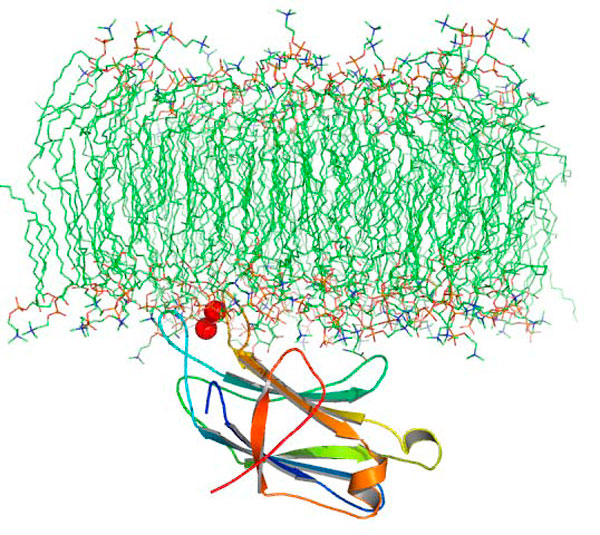
**An example of the peripheral domain (C2-domain of PKCα, PDB ID: **1DSY). The protein targets specific lipids in the membranes in response to certain signal which, in this case, is binding of 2 Ca^2+ ^ions (shown as red spheres). The protein (shown in cartoon representation) penetrates the membrane partially. Lipid hydrogens are not shown for clarity.

One of the limitations of supervised learning is that examples or instances from both the classes are required to build a classifier. Unavailability of sufficiently large set of examples from both classes is quite often the case with biological data due to various reasons: expenses and time required to obtain the data and other experimental limitations. Instead of having examples from both the classes, what is usually available is a sizeable set from one class and a much larger number of examples that are *unlabeled*. This is one of the most common occurrences in pharmaceutics and bioinformatics. For example, usually there are only very few inhibitors/drugs performing a certain function but a much larger number of drugs that have not been tested which would form the *unlabeled *set. This problem of unavailability of well-annotated examples from both the classes can be addressed by a special class of learning called *semi-supervised learning *or *partially supervised learning*. A recently developed approach to execute semi-supervised learning is the *Positive-Unlabeled (PU) learning *[2,3] using two sets: a well-defined *positive *set, and a much larger set with *unlabeled *examples. In this paper, we present the first implementation of *PU-learning *towards a bioinformatics problem: identification of peripheral domains that bind various membranes reversibly (Fig 1).

Peripheral proteins target different kinds of membranes (cellular, nuclear etc) in response to certain signals. These proteins, different from integral membrane proteins, are mainly cytosolic (Figure 1) [4] and also play crucial roles in membrane trafficking and in anchoring cytoskeletal structures. Their reversible attachment to biological membranes has been shown to regulate the biochemistry of the cell through a variety of mechanisms [5]. Many of these peripheral proteins have been directly or indirectly involved with many deadly diseases like cancer and AIDS [6,7]. In various kinds of human cancers, a common signal is the overproduction of a phospholipid, phosphatidylinositol (3,4,5) trisphosphate (PIP_3_), by the downstream action of AKT [6] that is activated by an interaction between PIP_3 _and a very common membrane-targeting domain called PH domain[7]. Similarly, during the late phase of HIV type 1 (HIV-1) replication, newly synthesized retroviral Gag proteins target the plasma membrane and interact with another phospholipid, phosphatidylinositol (4,5) bisphosphate (PIP_2_), an event that is essential for viral replication and pathogenesis [8]. Thus, the medical and molecular importance of these domains and their host-proteins is well established.

Structural biology has deciphered the structural basis of specific lipid binding and membrane interactions of membrane targeting domains and peripheral proteins. However, it would be prohibitively time-consuming and expensive to search and identify new peripheral proteins on a genomic-scale by these experimental approaches. Therefore, a fast and accurate bioinformatics-based annotation scheme would greatly supplement the effort to identify membrane-binding peripheral proteins on a genomic scale.

Although structural genomics projects progressively are gaining speed, it will still take many years before 3D structures of most proteins in the proteome of commonly studied organisms become available. So, for genome-wide prediction of peripheral proteins the need for sequence-based prediction protocols that don't rely on structures and can give reasonable precursor performance becomes inevitable. Here we broaden the prediction of peripheral proteins to that based on the features derived from their sequences alone to advance in the direction of whole-genome prediction.

In case of peripheral domains, there is a well defined set of domains which are known to bind the membranes with different affinities. However, there is a much larger set of proteins whose affinities have not been measured. So, there is no such fairly large dataset for the proteins that are known *not to bind membrane*. One of the common criteria for constructing a negative set is the subcellular localization. For example, the proteins that reside in the cytosol can not perform the DNA-binding function. However, this criteria can not be applied in this case as membranes confine many organelles of the cell (nucleus, mitochondria, the cell itself) and can be accessed anywhere. So, there is no golden negative set of proteins that are known not to bind membrane, thus standard supervised learning can not be applied here directly. So, here in this paper, we address this problem with the first bioinformatics application of *PU-learning *to identify peripheral domains based on their sequence-derived properties. We will show that *PU-learning *in combination with other auxiliary binary classification algorithms can be effectively used to build an identification protocol for predicting the membrane binding properties of a large number of modular domains with unknown properties.

Previously, there have been attempts to identify peripheral proteins using machine learning [9,10] that achieved an accuracy of about 90%. However, one major difference between those works and this study is that they were based on structural features of the proteins and so were based on a much smaller set of about 40 proteins with 3D structures. The negative set in those studies was also a high confidence one as those structures were well studied and annotated. In this study, on the other hand, we do not require structure of the proteins and use only sequence based descriptors so we have a much larger positive set and a poorly annotated negative set.

## Methods

In simple words, the machine learning problem being addressed here can be stated as: given a set of membrane-binding proteins, can we identify other membrane-binding proteins from a larger unlabeled set while classifying the proteins in the unlabeled set as positive or negative? The main components of the classification process are feature development, classifiers, validation techniques and performance criteria which are discussed below after explaining PU-learning in detail.

### Theory behind PU-learning

**Figure 2:**
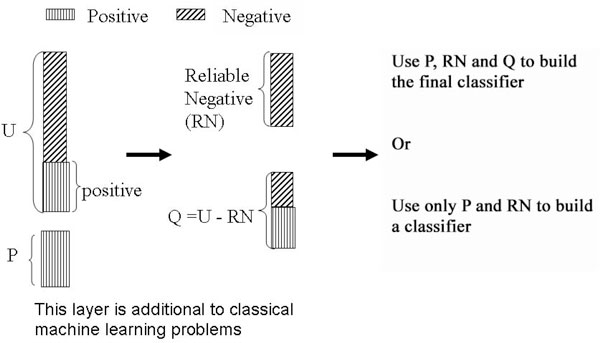
**Two step strategy used in *Positive-Unlabeled learning***. In the first step, a set of reliable negative examples are identified using a spy technique. During the spy technique, some positive examples are included in the *U *set as spies. After the classification, a threshold is chosen such that all the spies are classified as positive and the ones below the threshold form the reliable negative (*RN*) set. In the second step, the *RN *set and the *P *set are used to build a classifier.

*PU-learning *attempts to build a classifier using a two step-strategy [2,3] (Figure 2):

**Step 1**: Identifying a set of reliable negative examples from the unlabeled set.

**Step 2**: Building a set of classifiers by iteratively applying a classification algorithm and then selecting a good classifier from the set.

These two steps together can be seen as an iterative method of increasing the number of unlabeled examples that are classified as negative while maintaining the number of correctly classified positive examples. There are a couple of techniques proposed for each step. For the first step, Rocchio technique, the Spy technique, and the 1-DNF technique can be used. For the second step, any classifier, such as SVM, Bayes classifier, random forest or decision trees can be used. For the focus of this article, only the spy-technique is explained in great details. The readers are directed to relevant literature [2,3] for details about the other techniques regarding the first step.

**Figure 3:**
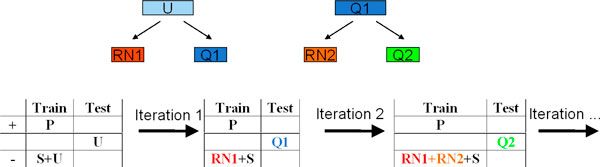
**The spy technique**.

In the spy technique, "spy" examples from the positive set (called the *P *set) are sent to the mixed or unlabeled set (called the *U *set) (Figure 3). This approach randomly selects *s*% of the examples from the *P *set (in our experiment, we use 15%). These examples form the 'spies' set, denoted by *S*, which is added to the *U *set. The spies behave identically to the unknown positive examples in *U *and hence allow us to reliably infer the behavior of the unknown positive examples. The algorithm is as follows:

1. N = •

2. S = sample (P, s%)

3. U = U ∪ S

4. P = P - S

5. Assign every example in P the class c1

6. Assign every example in U the class c2

7. Run any classifier

8. Classify each example in U

9. Determine the probability threshold t using S

10. for each example dj in U

11.   if its probability Pr [c1|dj] < t

12.      RN = RN ∪ {dj}

13.      U = U - {dj}

14. Repeat steps 7 to 13 with RN and U until RN does not change

The first classifier is built using the *P *set (after removal of spies) as positive set and the *U *+ *S *(spies set) as the negative set. This classifier is then tested on the *U+S *set. A threshold is then determined such that all the spies are classified as positive. The unlabeled examples that are below that threshold form the first reliable negative (*RN1*) set and the remaining examples in *U *form the *Q1 *set. The process is then repeated with *P *(combined with *S *set) as positive and the reliable negative (*RN*) set as negative and the resultant classifier is tested on the *Q *set to further extract reliable negative examples from the *Q *set. The process above is repeated until no more examples in the *Q *set can be classified as negative. The final classifier is then built using the *N *and the original *P *set. The pseudo code is as follows:

Every example in P is assigned the class label 1;

Every example in RN is assigned the class label -1;

i = 1;

Loop

**Figure 4:**
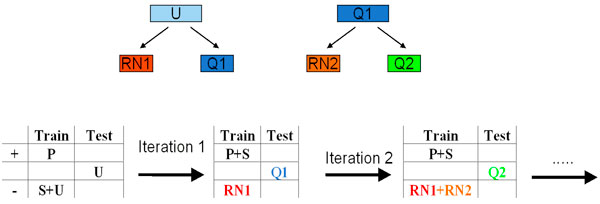
**Modified spy technique**.

   Use P and RN to train a classifier Si;

   Classify Q using Si;

   Let the set of examples in Q classified as negative be W

   if W = {} then exit-loop

   else Q = Q - W;

      RN = RN ∪ W;

i = i+1;

In this article, we also propose and implement a variation of the spy-technique. The spies are added in each iteration as opposed to just the first iteration [3]. Figure 4 shows this technique in a cartoon representation.

So, *PU-learning *adds an additional layer to the standard supervised learning. In this layer the first set of reliable negative examples is created. Using this reliable negative set and the original positive set, more reliable negative examples are extracted in the subsequent steps using a classifier iteratively. For this study, spy-technique was used for the first step and random forests were used for the second step. As we will show, the *PU-learning *protocol with spy-technique can be effectively used to build an identification protocol for predicting the membrane binding properties of a large number of modular domains with unknown properties.

### Dataset

For the creation of positive dataset, entire human, mouse and yeast proteomes were downloaded from the Swiss-Prot database [11]. All the sequences of the peripheral domains were then extracted by using their names as keywords resulting in 932 cases. The known domains include C1, C2, PH, PX, FYVE, ANTH, BAR, FERM and Tubby domains. Sequence identity was then reduced to 40% using CD-HIT [12] among all the pairs reducing the number of sequences to 232. For unlabeled set, all the other domains except the positive ones were selected from the three proteomes giving a total of approximately 32,000 examples. After reducing the sequence identity to 20%, the number of unlabeled examples was 3,759. A higher sequence identity was used for the positive case as the number of positive examples is few and using a lower sequence identity would result in much fewer examples that might be insufficient for building a reliable classification model.

### Features

During feature development, a protein sequence is reduced to a fixed set of features encoding the characteristics of a protein. It is always advisable to choose the features that are supposed to be pertinent to the function of a protein and display large variation between positive and negative set.

All intracellular membranes contain varying degrees of anionic lipids with the inner plasma membrane being the most anionic [13,14]. Thus, electrostatic complementarity between cationic proteins and anionic membranes should be an important factor in membrane binding of peripheral proteins. Thus, on the basis of previous studies on membrane binding proteins, various sequence-based features were selected: the overall charge of the protein; the sum of hydrophobicity, helix propensity and sheet propensity; and the overall sequence composition of the domain (% of each kind of amino acid). In addition, a new family of features called local environment amino acid composition is also used. This feature representation defines a residue based on both its identity and its environment of found kinds: low helix and high sheet propensity, high helix and low sheet propensity, and so on. This forms 80 (4 × 20) distinct counts in the new feature vector.

### Performance criteria and evaluation technique

The performance of the classifiers is measured using different metrics. Specifically, the commonly-used threshold metrics include accuracy and sensitivity. Accuracy is the ratio of correct predictions to the total number of predictions.(1)

Sensitivity, also known as recall or true positive rate, TPR, is defined as the probability that a prediction is predicted positive given the example *is *positive. It is approximated by the fraction of true positives predicted as positive.(2)

For evaluating the performance of the protocol, the holdout technique was used during testing. During this evaluation, 40 positive examples were left out for testing the final classification protocol. These examples were not used for training purposes at any stage of the model building. Due to an uncertainty about their classes, no examples from the unlabeled set were left out for testing and so only sensitivity was used for performance evaluation. During training, 5-fold cross-validation was used to optimize the parameters.

### Classifier

Decision trees, specifically, C4.5 was used as a classifier. A decision tree [15] constructs from the training data a tree model where every internal node represents a decision and a leaf represents its classification. The learning process starts by finding a split on a single attribute that best classifies the training data; then the dataset is recursively split into two parts repeating these steps on each subset. There are a number of loss (or impurity) functions that are used to find the best split or the split with the minimum loss (or error). Specifically, the C4.5 [16] decision tree algorithm developed by Quinlan uses a loss function known as the information gain, which is motivated by information theory. The decision tree has several advantages. First, it is fast to train and evaluate. Second, the model (or function) learned during the training process is usually compact and easy to interpret. Finally, a decision tree does not require much data preprocessing, natively handling most attributes types. Note, most machine learning algorithms have tunable parameters. In this work, the results reported using the C4.5 decision tree algorithm use the default values empirically found to work well on a number of datasets.

## Results

During the initial iteration, the first reliable negative set is created by the examples that were classified as negative (those that fell below the threshold of the spies) from the unlabeled set and the remaining unlabeled data form the *Q *(= *U*-*RN1*) set. In subsequent iterations, some examples from the *Q *set are identified as reliable negative and are added to the *RN *set. Thus, the *RN *set keeps expanding with the addition of more proteins from the unlabeled set while the *Q *set keeps shrinking. This process is repeated until no examples from the *Q *set can further be classified as negative.

**Figure 5:**
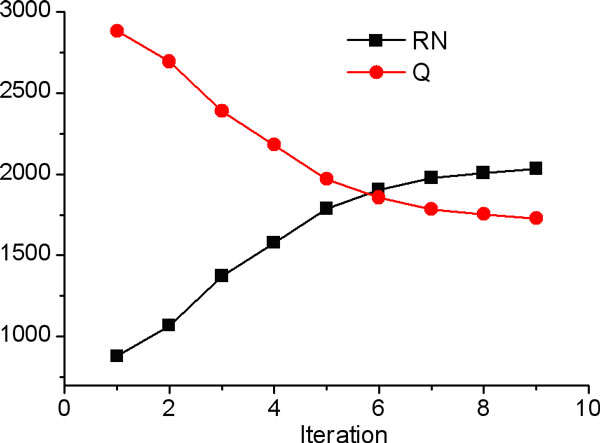
**Reliable negative (*RN*) and the *Q *(= *U-RN*) sets as a function of iteration with the spy technique**.

We selected 30 positive examples (approximately 15% of total 232 positive cases) and added them to the unlabeled set as spies carrying a negative label (*U+S*). The *U+S *set and the remaining 202 positive cases were then used to build the first binary classification protocol that was tested on the *U+S *set. Based on the threshold so that all the spies are classified as positive, 877 examples were identified from the *U *set as reliable negative examples (*RN1 *set). With this *RN1 *set and the initial positive examples, more reliable examples were extracted from the *Q *set until no more examples in *Q *could be classified as negative. Figure 5 shows the growth of the reliable negative set and the shrinking of the *Q *(= *U-RN*) set. After 10 iterations, both *RN *and *Q *set converged at 2,031 and 1,728 examples.

This final model built in the last iteration was then used to classify each example in the holdout set composed of 40 positive examples. 38 of these 40 examples were classified as positive and only 2 were classified as negative giving an accuracy of 95% with a sensitivity of 95%. When compared with the fraction of *Q*, which is only 46% of the total dataset, this 95% sensitivity in testing set shows that the classification protocol learnt to identify the positive examples with a high accuracy.

**Figure 6:**
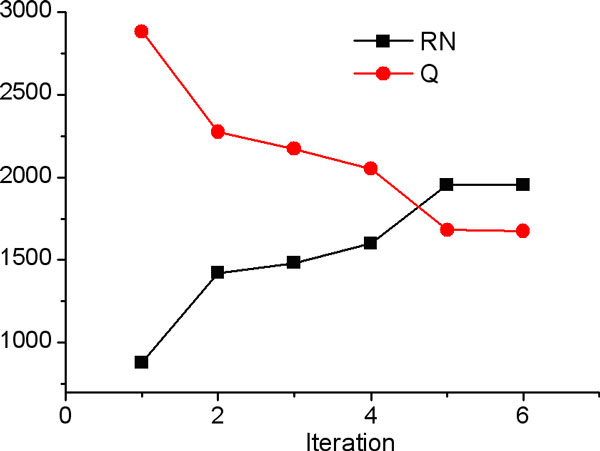
**Reliable negative (*RN*) and the *Q *(= *U-RN*) sets as a function of iteration with the modified spy technique**.

Another variation of the spy technique was also tried: the spies were added during each iteration as opposed to just the first iteration implemented before. Figure 4 shows this technique in a cartoon representation. Similar procedure as above was then performed for this spy technique. Similar to the first implementation, with each iteration, *RN *set grows and *Q *set shrinks until convergence. Figure 6 shows the trend of the two set as a function of iteration. The protocol converged with the same set of *RN *examples as in the first implementation increasing the confidence in this set. With this modification of the spy technique, the performance of the protocol was evaluated, as above, by testing the final model on the holdout test of 40 proteins. Similar to the first case, 38 domains were correctly classified and only 2 were incorrectly classified giving an accuracy of 95% and a similar sensitivity. The two proteins that were misclassified were the same misclassified proteins as in the first implementation: *Protein pob1 *and *Oxysterol-binding protein 1*. Upon further examination, we found that these proteins have very dissimilar characteristics such as amino acid and dipeptide composition to other positive cases. This is also demonstrated by the fact that both these proteins share less than 15% and 16% sequence identity, respectively, with any other sequence in the set. Performances of both the protocols show their potential for genome-scale identification of peripheral domains.

## Discussion

One of the common issues in building machine learning based classifiers is the unavailability of reasonably large sets of examples from both (or all) the classes in case of binary (or multi) class problems. This problem of having partial information is all the more encountered in biological fields where collection of data may not be an easy task. So, what is usually available is a set of examples belonging to one of the two classes and a much larger set of unlabeled examples that might belong to either class. The task of building a classifier with this partial information then falls under semi-supervised learning and can be accomplished through *Positive-Unlabeled *learning.

Here in this article, we implemented a classification protocol that represents the first attempt to identify and predict membrane-binding domains on the basis of their sequence-derived features using *positive-unlabeled *learning. The application of *PU learning *surpasses the unavailability of a well-define negative set consisting of proteins that are known *not to bind membranes*. This is, to the best of our knowledge, the first application of *PU-learning *to any bioinformatics problem. Prior to this work, it has only been applied to text classification of web pages [2,3].

We have used simple sequence-derived features of known peripheral proteins to identify other putative membrane-binding proteins also proposed a list of other proteins that, we believe, are highly unlikely to bind membranes. Simple sequence-derived features are used to build the protocol. Two independent tests demonstrate that the developed protocol can identify peripheral domains with a high accuracy. In both the tests, accuracy achieved was 95% whereas a similar sensitivity was registered. These results combined with the fact that this protocol only uses sequence-derived features without actually depending on sequence homology suggest that our protocol can be used to predict the membrane-binding properties of modular domains, including other membrane targeting domains, with high accuracy. For those proteins without modular domains and homology to known proteins, our protocol can serve as initial screening for potential candidates.

In this article, we also propose a modified spy technique where the spies are added to the unlabeled set in each iteration instead of just the first one. Each time, a randomly selected spy set is added that makes the protocol more robust. When the *Q *set converges even after adding a new set of spies during each iteration, it renders more confidence in the final reliable negative (*RN*) set. Also, the fact that the final RN set was the same in both implementations (original and modified spy technique) also boosts the reliability of the protocol's prediction.

As an output of this protocol, we have proposed two sets: a set of proteins that are most unlikely to bind membranes (final *RN *set) of ~2000 proteins and the remaining *Q *set of ~1700 proteins that can not be classified clearly in either class. Proteins belonging to both *RN *and *Q *set were picked by the protocol from the initial unlabeled set of ~3700 set. So, essentially, we have reduced the number of proteins which are expected to bind membranes to a representative set of ~1700 which would provide a good initial list of proteins to test experimentally for their membrane binding properties for a genome-wide identification of membrane-binding proteins. Efforts and collaborations are underway to test the *Q *set to test for their membrane-binding properties (Professors Wonhwa Cho and Robert Stahelin, personal communication).

It should be noted here that a limitation of the method employed above, and of generative machine learning in general, is that it identify new instances on the basis of features learnt from training examples; identification of novel instances that are not similar to any the training instances is difficult. This is particularly the case for peripheral proteins as they use a variety of features to target membranes. So, to partially overcome this limitation, we introduce as much variety in our training/spy set as possible with the given data by using a modified version of PU learning where we randomly use a new spy set in each iteration.

## Conclusion

In this work we applied PU learning to a protein function prediction problem: identifying membrane binding domains. Encouragingly, the high accuracy prediction (at 95%) of the holdout set share very low sequence identity with the training set; 50% of the pairs between these sets have only 6% identity and all the pairs were less than 20% identical. These identities are much lower than the limit for identifying similar cases based on homology searches. This shows that PU method performs better than just identifying novel instances based on sequence homology.

In general, the above protocol can be applied to any bioinformatics problem where a similar issue is encountered. For example, this protocol will especially prove useful in problems like prediction of protein-protein interactions (where a golden negative set of proteins that are *known not to interact with each other *is not available) and quantitative structure-activity relationship (QSAR) studies where information about the negative set of proteins not related with a biological or chemical activity is highly scarce. We believe in present times when our knowledge is limited and ever-increasing, protocols like the one presented in this article that can make use of only partial information will prove to be very useful.

## Competing interests

The authors declare that they have no competing interests.

## Authors' contributions

NB conceived of the study, carried out the data analysis and helped in drafting the manuscript. MG participated in the design of the study and helped in drafting the manuscript. HL conceived of the study, and participated in its design and coordination and helped to draft the manuscript. All authors read and approved the final manuscript.
